# Феохромоцитома при нейрофиброматозе 1 типа

**DOI:** 10.14341/probl13608

**Published:** 2026-03-07

**Authors:** Д. В. Реброва, О. И. Логинова, С. Л. Непомнящая, А. Р. Бахтиярова, В. Ф. Русаков, Л. М. Краснов, Е. А. Федоров, И. К. Чинчук, Ш. Ш. Шихмагомедов, Е. Н. Имянитов, О. В. Кулешов, М. А. Алексеев, Т. С. Придвижкина, Т. В. Савельева, А. А. Семенов, Е. А. Згода, Р. А. Черников, И. В. Слепцов

**Affiliations:** Санкт-Петербургский государственный университет, Клиника высоких медицинских технологий им. Н.И. ПироговаРоссия; Saint Petersburg State University, Saint Petersburg State University HospitalRussian Federation; Санкт-Петербургский государственный университет, Клиника высоких медицинских технологий им. Н.И. Пирогова; Национальный медицинский исследовательский центр им. В.А. АлмазоваРоссия; Saint Petersburg State University, Saint Petersburg State University Hospital; Almazov National Medical Research CenterRussian Federation; Национальный медицинский исследовательский центр онкологии им. Н.Н. ПетроваРоссия; National Medical Research Center of Oncology n.a. N.N. PetrovRussian Federation

**Keywords:** новообразование надпочечника, инциденталома надпочечника, феохромоцитома, болезнь Реклингхаузена, нейрофиброматоз 1 типа, adrenal tumor, adrenal incidentaloma, pheochromocytoma, Recklinghausen’s disease, neurofibromatosis type 1

## Abstract

Нейрофиброматоз 1 типа — наследственное заболевание с широкой вариабельностью клинических проявлений, от практически полного отсутствия типичных симптомов до мультисистемного поражения организма. Одним из возможных клинических проявлений данной патологии является феохромоцитома — опухоль надпочечника с возможным развитием грозных осложнений со стороны сердечно-сосудистой системы. В статье представлено описание четырех случаев пациентов с феохромоцитомой в составе семейного нейрофиброматоза 1 типа, различающихся по клиническому течению от бессимптомной формы до ярких пароксизмальных проявлений. При этом наличие и степень артериальной гипертензии не коррелировали с уровнем метанефринов и размерами феохромоцитомы. У трех из четырех пациентов установлен наследственный анамнез нейрофиброматоза 1 типа. В одном из четырех случаев отмечалось симультанное двустороннее поражение надпочечников, при этом лучевые характеристики феохромоцитом, как при компьютерной томографии, так и при КТ/ПЭТ с 18-ФДГ, отличались от «классических». Объективный осмотр с выявлением «стертых» признаков нейрофиброматоза 1 типа позволили установить диагноз двусторонней феохромоцитомы даже при сомнительных лабораторных и визуализирующих данных. Знание клинических проявлений, своевременная диагностика нейрофиброматоза 1 типа, комплексное лечение и последующее регулярное наблюдение пациентов, а также обследование кровных родственников позволяют в значительной степени улучшить прогноз и выживаемость.

## Abstract (en)

Neurofibromatosis type 1 is a hereditary disease with a wide variability of clinical manifestations, from the almost complete absence of typical symptoms to a multisystem lesion of the body. One of the possible clinical manifestations of this pathology is a pheochromocytoma — a tumor of the adrenal gland with the possible development of considerable cardiovascular complications. The article describes four cases of patients with pheochromocytoma as part of familial neurofibromatosis type 1, differing in clinical course from asymptomatic form to vivid paroxysmal manifestations. At the same time, the presence and degree of arterial hypertension did not correlate with the level of metanephrines and the size of the pheochromocytoma. 3 out of 4 patients have a hereditary history of neurofibromatosis type 1. In 1 out of 4 cases, simultaneous bilateral damage to the adrenal glands was noted, while the radiation characteristics of pheochromocytomas, both with computed tomography and CT/PET with 18-FDG, differed from the "classic" ones. An objective examination with the identification of "erased" signs of neurofibromatosis type 1 made it possible to establish the diagnosis of bilateral pheochromocytoma even with questionable laboratory and imaging data. Knowledge of clinical manifestations, timely diagnosis of neurofibromatosis type 1, comprehensive treatment and subsequent regular monitoring of patients, as well as examination of blood relatives can significantly improve prognosis and survival.

## АКТУАЛЬНОСТЬ

Нейрофиброматоз 1 типа (НФ1) — генетически-детерминированное заболевание с аутосомно-доминантным типом наследования, вызванное мутациями зародышевой линии в гене-супрессоре опухоли NF1 и характеризующееся развитием новообразований различных органов и систем [1]. Распространенность данного наследственного синдрома составляет 1:1900–1:3500 населения [2].

У носителей мутации NF1 отмечаются характерные патологические изменения на коже в виде пятен цвета «кофе с молоком», веснушчатой гиперпигментации и нейрофибром [3]. У больных НФ1 могут выявляться новообразования нервной системы, такие как глиомы зрительного нерва, спинальные нейрофибромы, плексиформные нейрофибромы, доброкачественные и злокачественные опухоли оболочек периферических нервов с возможным развитием компрессионного синдрома [4]. Кроме того, в данной группе отмечаются трудности с обучением, дефицит внимания, а также социальные и поведенческие проблемы. Со стороны опорно-двигательной системы нередко наблюдаются сколиоз, псевдоартроз в области большеберцовой кости, дисплазия орбит [5][6]. Сообщается по повышенном риске рака молочной железы у женщин с НФ1 [2]. Эндокринная патология, ассоциированная с мутацией гена NF1, включает феохромоцитому/параганглиому (ФХЦ/ПГ) и другие новообразования надпочечников, гастроэнтеропанкреатические нейроэндокринные опухоли, узлы щитовидной железы различной природы [7].

НФ1 известен также как болезнь Реклингхаузена (Von Recklinghausen’s disease, Von Recklinghausen neurofibromatosis), потому что впервые подробно был описан в монографии «Множественные фибромы кожи и их связь с множественными невриномами» Дж. фон Реклингхаузена, вышедшей в 1882 г. [5]. Однако первые критерии заболевания были установлены лишь в 1987 году, что связано с разнообразием клинических проявлений наследственного синдрома даже у лиц из одной семьи с идентичной мутацией [1]. Предполагается, что одной из основных причин фенотипического полиморфизма заболевания у больных НФ1 является появление дополнительных соматических мутаций непосредственно в опухолях, что может затруднять диагностику, особенно в бессимптомных случаях [8].

НФ1 возникает в результате мутации в гене опухолевого супрессора NF1, расположенного на 17 хромосоме (17q11.2) [5]. Около половины случаев заболевания связаны со спорадической мутацией [7]. Аутосомно-доминантный тип наследования с полной пенетрантностью гена являются причиной включения наличия родственника первой линии родства в критерии постановки диагноза [9].

В большинстве случаев НФ1 можно установить на основании клинических симптомов без проведения генетического исследования. Критериями диагностики является сочетание двух и более признаков из нижеперечисленных [10]:

Однако постановка диагноза может быть затруднена у маленьких детей (до 6 лет), а также у детей с мутацией NF1 de novo (без семейного анамнеза), у которых пятна цвета «кофе с молоком» на кожных покровах являются единственным клиническим признаком. В таких случаях может потребоваться генетическое тестирование для подтверждения диагноза НФ1 [11].

Одним из характерных новообразований, возникающих при НФ1, является ФХЦ/ПГ — это нейроэндокринная опухоль, состоящая из хромаффинных клеток, продуцирующая катехоламины (адреналин, норадреналин и дофамин) [12]. Феохромоцитома (ФХЦ) является частным случаем симпатической параганглиомы (ПГ), локализованная в мозговом слое надпочечников [7]. ПГ не характерны для НФ1 и встречаются крайне редко [13]. Распространенность ФХЦ при НФ1, по оценкам разных авторов, составляет 0,1–7,7%, при этом по данным аутопсий она возрастает до 13–14,6%, а при сочетании с артериальной гипертензией (АГ) может достигать 20–50% [2][6][7][14][15][16]. Неоднозначны взгляды на распространенность бессимптомного течения ФХЦ при НФ1. В одних источниках сообщается о 22% случаев [2][17], тогда как в других описано более 80% [18]. Подобные различия могут быть связаны с малым количеством пациентов в исследуемых выборках. При этом неоднократно было показано влияние гиперкатехоламинемии на состояние сердечно-сосудистой системы даже в отсутствие клинических проявлений [19][20]. Важно, что в ряде исследований сообщается о высоком риске метастазирования ФХЦ при НФ1, составляющем около 12% [18][21][22]. В настоящее время Европейская целевая группа по генетическим синдромам с опухолевым риском (ERN GENTURIS) рекомендует проводить скрининг на ФХЦ у лиц с НФ1 при повышении уровня артериального давления (АД) без других выявленных причин, при любом планировании оперативного лечения с наркозом у взрослых, а также у всех женщин при планировании или наступлении беременности [23].

## Описание клинических случаев

## Клинический случай №1

Пациентка Н., 58 лет, обратилась в Клинику высоких медицинских технологий им. Н.И. Пирогова Санкт-Петербургского государственного университета (КВМТ СПбГУ) с жалобами на приступы повышения уровня АД до 200/120 мм рт.ст., сопровождающиеся учащенным сердцебиением, головной болью давящего характера, часто — носовыми кровотечениями.

Из анамнеза известно, что периодическое повышение уровня АД до 140–160/90–100 мм рт.ст. появилось после 50 лет, однако в последние полгода АГ приобрела кризовый характер. Вышеописанные приступы развиваются преимущественно в ночные часы, купируются самостоятельно в течение 2–3 минут. Пациентка сообщила о перенесенных в детстве четырех операциях по поводу расходящегося косоглазия со слабоположительным клиническим эффектом, а также об удалении кисты спинного мозга в 27 лет (выписные справки представлены не были).

С целью исключения вторичных причин АГ направлена кардиологом на ультразвуковое исследование (УЗИ) органов брюшной полости (ОБП) и почек, при котором выявлено новообразование левого надпочечника. При компьютерной томографии (КТ) выявленная опухоль имела размеры 42х45х49 мм, нативную плотность от +15 до +50 HU, отмечалось кистозно-солидное строение за счет мягкотканного компонента по периферии и кистозного компонента в центральных отделах, визуально с наличием эффекта седиментации. По данным лучевых характеристик были заподозрены ФХЦ или адренокортикальный рак. При лабораторном обследовании с целью определения гормональной активности новообразования надпочечника установлено значимое увеличение экскреции метаболитов катехоламинов, преимущественно за счет общего метанефрина — 4365 мкг/сут (до 400), с небольшим повышением уровня общего норметанефрина до 932 мкг/сут (до 900). По результатам ночного теста с 1 мг дексаметазона уровень кортизола составил 106,8 нмоль/л, что соответствует «серой зоне». Таким образом, установлена феохромоцитома левого надпочечника с возможной автономной гиперпродукцией кортизола.

Объективно: рост 164 см, вес 60 кг. При осмотре обращали на себя внимания наличие множественных пигментных пятен цвета «кофе с молоком», узелковых новообразований кожи от 4 до 15 мм в диаметре, веснушчатость всего тела, сколиоз грудного отдела позвоночника. Пациентка Н. сообщила, что первые признаки кожных проявлений отметила в 26 лет, во время беременности, с дальнейшим постепенным распространением практически по всем кожным покровам (рис. 1). Наличие подобных кожных проявлений у матери отрицала. Знала, что мать длительно страдала гипертонической болезнью, умерла от инсульта в 65 лет. Уточнить наследственный анамнез по отцовской линии не удалось, так как жили отдельно и длительно не общались. У пациентки есть дочь, которой на момент операции было 32 года, без характерных кожных проявлений.

**Figure:**
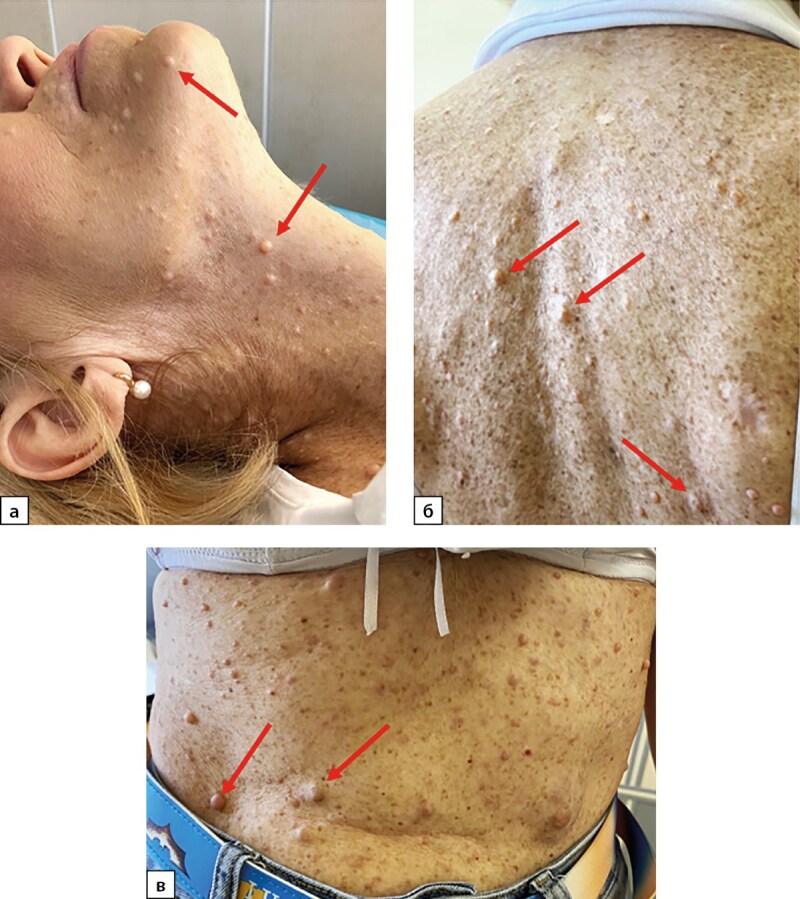
Рисунок 1. Кожные проявления нейрофиброматоза 1 типа у пациентки Н., 58 лет. а — нейрофибромы кожи лица и шеи, б — нейрофибромы и «веснушчатая», гиперпигментация кожи спины, в — нейрофибромы и «веснушчатая» гиперпигментация кожи живота. Стрелками обозначены нейрофибромы кожи.

Учитывая клинические признаки НФ1, проведено дополнительное обследование. При УЗИ щитовидной железы (ЩЖ) выявлены узлы в правой доле — 12х7х9 мм и 7х4 мм, в левой доле — узлы 4х4 мм, 4х2 мм, а также множественные образования по 3 мм. Кроме того, позади левой доли ЩЖ визуализировано гипоэхогенное неоднородное образование 16х5х9 мм. Лабораторно уровень тиреотропного гормона (ТТГ) составил 3,36 мкМЕ/мл (0,4–4,0), кальцитонина — 3,53 пг/мл (до 4,8), общего кальция — 2,54 ммоль/л (2,15–2,58), ионизированного кальция — 1,27 ммоль/л (1,16–1,32), паратгормона — 69,98 пг/мл (15–65). Выполнена тонкоигольная аспирационная биопсия (ТАБ) гипоэхогенного узла правой доли ЩЖ размером до 12 мм — цитологическая картина коллоидного узла (Bethesda II), а также гипоэхогенного объекта в нижней яремной зоне слева — материал неинформативный (Bethesda I), в смывах с иглы тиреоглобулин и паратгормон не определялись, сделан вывод о том, что данное образование является лимфатическим узлом. С учетом отсутствия первичного гиперпаратиреоза в составе синдрома НФ1 незначительное повышение уровня паратгормона расценено как вторичное, вероятно, на фоне дефицита витамина D, в связи с чем инициирована терапия холекальциферолом в дозировке 4000 МЕ в сутки.

При магнитно-резонансной томографии (МРТ) головного мозга описана картина единичного супратенториального очага глиоза, что расценено как проявление микроангиопатии.

С целью подготовки к оперативному лечению инициирована терапия доксазозином с постепенным увеличением дозировки до 8 мг в сутки, добавлением к схеме лечения бисопролола в дозе 1,25 мг в сутки. На этом фоне было достигнуто урежение приступов АГ не выше 130/90 мм рт.ст., пульсом до 80 ударов в минуту, уровнем АД в межприступный период 90–110/60–70 мм рт.ст.

По семейным обстоятельствам госпитализация с целью оперативного лечения была выполнена только через 7 месяцев после установления диагноза ФХЦ. При контроле КТ выявлено увеличение новообразования левого надпочечника в размерах до 61х49х53 мм, с кистовидной толстостенной структурой, с толщиной капсулы 3–5 мм, плотностью ткани капсулы +30 HU, накоплением контрастного вещества (КВ) максимально до +55 HU, выведением через 10 минут до +45 HU (рис. 2). На фоне целевого уровня 25ОН-витамина D 36,76 нг/мл (30–100) отмечена нормализация уровня паратгормона — 38,44 пг/мл (15–65), нормокальциемия. При суточном мониторировании электрокардиографии (ЭКГ) по Холтеру выявлена желудочковая экстрасистолия I градация по Ryan.

**Figure:**
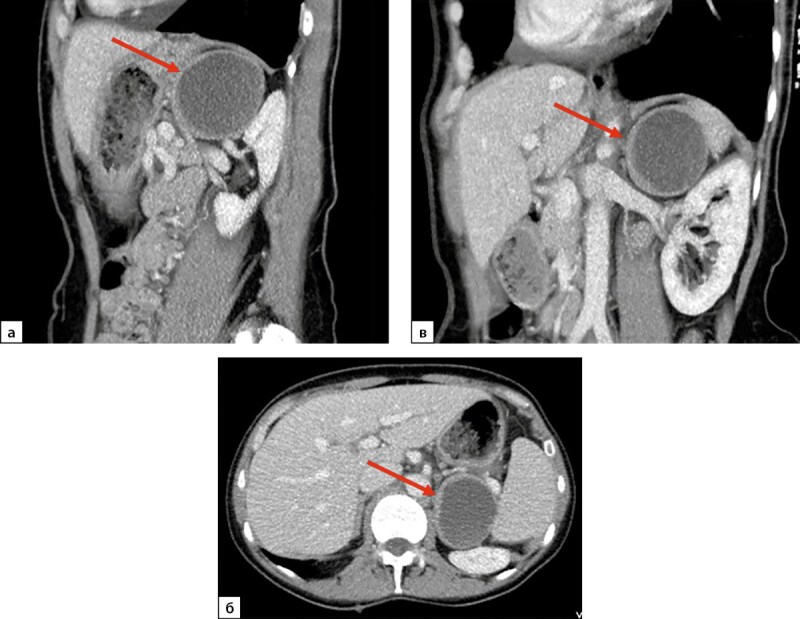
Рисунок 2. Компьютерная томография надпочечников пациентки Н., 58 лет, артериальная фаза. а — сагиттальная плоскость, б — аксиальная плоскость, в — фронтальная плоскость. Стрелками обозначена феохромоцитома левого надпочечника, хорошо визуализируется симптом «кольцевого ободка».

Пациентке была проведена однопортовая ретроперитонеоскопическая адреналэктомия слева. По результатам гистологического исследования подтверждена ФХЦ с вторичными изменениями, без сосудистой и капсулярной инвазии.

В послеоперационном периоде в связи с клиническими признаками надпочечниковой недостаточности к терапии добавлен гидрокортизон 45 мг в сутки с дальнейшим постепенным снижением дозировки.

Пациентке было рекомендовано расширенное генетическое тестирование, однако она выполнила исследование на носительство мутации RET-протоонкогена, при котором в 16 экзоне RET был выявлен гетерозиготный вариант с.2777А>T (p.His926Leu), не описанный в доступных базах данных и литературе (вариант RET с неизвестным клиническим значением).

После адреналэктомии в течение четырех лет пациентка Н. проходит ежегодное обследование. В настоящее время послеоперационная надпочечниковая недостаточность компенсирована приемом 7,5 мг гидрокортизона в сутки. Данных за рецидив ФХЦ не получено.

## Клинический случай №2

Пациентка Ш., 22 года, поступила Клинику высоких медицинских технологий им. Н.И. Пирогова Санкт-Петербургского государственного университета (КВМТ СПбГУ) с целью оперативного лечения. На момент обращения жалоб активно не предъявляла. Уровень АД при неоднократных амбулаторных измерениях и контроле в стационаре находился в пределах 110–125/65–75 мм рт.ст.

Из анамнеза известно, что в связи с наличием НФ1 типа у обоих родителей, клиническими признаками данного наследственного синдрома у пациентки проведено КТ ОБП, при котором выявлено новообразование правого надпочечника размерами до 29х31 мм, нативной плотностью до 36 HU, с повышением плотности при контрастировании максимально до 70 HU.

При лабораторном обследовании с целью определения гормональной активности новообразования надпочечника установлено повышение экскреции метанефринов с суточной мочой до 709 мкг (до 320) при нормальном уровне норметанефринов — 371 мкг/сут (до 900). Одновременно выявлено повышение уровня хромогранина А до 120,2 мкг/л (до 100), что в сочетании с данными лучевых и лабораторных методов исследования свидетельствовало в пользу ФХЦ. Уровень кортизола в крови был в норме и составил 384 нмоль/л (185–624), экскреция свободного кортизола не увеличена — 439,35 нмоль/сут (160–1002). Обращало на себя внимание повышение уровней кальцитонина до 5,03 пг/мл (до 4,8) и паратгормона до 13,4 пмоль/л (2–9,4) на фоне нормального уровня ионизированного кальция крови — 1,27 ммоль/л (1,13–1,31), при эутиреодном статусе пациентки — ТТГ 2,2 мМЕ/л (0,35–4,94), повышенном титре антител к тиреопероксидазе (АТ к ТПО) до 240,6 МЕ/мл (до 30). Уровень 25ОН-витамина D не оценивался. При УЗИ щитовидной отмечались диффузные изменения тиреоидной ткани, данных за аденому околощитовидной железы не получено. Гиперпаратиреоз расценен как вторичный на фоне дефицита витаминаvD, назначены препараты альфакальцидола. С целью подготовки к плановому оперативному лечению инициирована терапия доксазозин в дозе 1 мг в сутки.

По семейным обстоятельствам пациентка госпитализирована повторно с целью оперативного лечения только через 6 месяцев. На фоне приема доксазозина уровень АД сохранялся стабильным, пульс в покое 64–76 ударов в минуту. Около 2 раз в месяц отмечала легкие проявления ортостатической гипотензии. В Клинике высоких медицинских технологий им. Н.И. Пирогова Санкт-Петербургского государственного университета (КВМТ СПбГУ) выполнена ретроперитонеоскопическая адреналэктомия справа. По результатам гистологического исследования подтверждена ФХЦ правого надпочечника размером до 50 мм.

При контроле в послеоперационном периоде данных за надпочечниковую недостаточность получено не было: уровни адренокортикотропного гормона (АКТГ) и кортизола находились в пределах референсных значений, составив 7,63 пмоль/л (1,034–10,736) и 243,45 нмоль/л (185–624) соответственно. Электролиты крови были в норме: калий — 3,77 ммоль/л (3,5–5,3), натрий — 136,6 ммоль/л (135–148), кальций ионизированный — 1,29 ммоль/л (1,13–1,31). Уровень кальцитонина стал пограничным — 4,87 пг/мл (до 4,8), концентрация паратгормона снизилась до 10,2 пмоль/л (2–9,4).

При контрольной МРТ ОБП через полгода данных за рецидив ФХЦ получено не было. Дополнительно проведена МРТ головного мозга, при которой в области левой ножки мозга выявлен очаг гиперинтенсивного сигнала с гипоинтенсивным центром на Т2 размерами 9х8 мм округлой формы, с четкими контурами, без признаков компрессии окружающих структур. Была рекомендована консультация нейрохирурга. Дальнейшая связь с пациенткой была потеряна.

## Клинический случай №3

Пациент О., 34 года, поступил в Клинику высоких медицинских технологий им. Н.И. Пирогова Санкт-Петербургского государственного университета (КВМТ СПбГУ) с жалобами на эпизодическое повышение уровня АД максимально до 140/90 мм рт.ст. («привычное» АД 120–80 мм рт.ст.) и периодические боли в поясничной области.

Из анамнеза известно, что при обследовании по месту жительства по поводу болей в поясничной области при УЗИ почек выявлены новообразования обоих надпочечников, которые подтверждены результатами МРТ ОБП. При МРТ выявленные опухоли имели схожие характеристики с ровными, местами нечеткими контурами, однородно накапливающие парамагнетик, размерами слева 18х10х16 мм, справа – 19х16х14 мм.

Амбулаторно проведено лабораторное обследование с целью определения гормональной активности новообразований надпочечников. Данных за гиперальдостеронизм и гиперкортицизм получено не было: уровень альдостерона плазмы — 110 пг/мл (17,6–392), ренина — 60,3 мкМЕ/мл (2,8–46,1), альдостерон-рениновое соотношение (АРС) — 1,8 (до 12); экскреция кортизола с суточной мочой — 240 нмоль/л (до 485,6). В суточной моче выявлено незначительное повышение экскреции общего метанефрина до 439,3 мкг/сут (38–404) при нормальном выделении общего норметанефрина 282,9 мкг/сут (75–740). При этом уровень хромогранина А в крови находился в пределах референсных значений — 46,37 мкг/л (до 100).

Пациент консультирован дистанционно специалистами КВМТ СПбГУ с целью уточнения диагноза рекомендовано дообследование. В повторном анализе суточной мочи концентрация общего метанефрина составила 596,7 мкг/сут (38–404) при стабильно нормальном выделении общего норметанефрина — 315,9 мкг/сут (75–740). Анализ плазмы на метанефрины не выполнен ввиду его недоступности по месту жительства. Уровень 17ОН-прогестерона был в пределах нормы — 0,98 нг/мл (0,5–2,1). После пробы с 1 мг дексаметазона уровень кортизола снизился до 25,02 нмоль/л — получено полное подавление, исключен органический гиперкортицизм.

Учитывая двустороннее поражение надпочечников, подозрительное на ФХЦ, проведено лабораторное обследование с целью исключения синдрома множественной эндокринной неоплазии 2 типа (МЭН2) — все показатели находились в пределах референсных значений: ТТГ — 3,14 мкМЕ/мл (до 5), кальцитонин — 5,1 нг/мл (до 8,4), паратгормон — 3,5 пг/мл (1,16–7,58), кальций ионизированный — 1,14 ммоль/л (до 1,31).

При КТ с контрастированием в правом надпочечнике визуализировано новообразование размерами 20х16,8х11,3 мм и нативной плотностью до 47 HU, с накоплением контрастного вещества в артериальную фазу до 74-94 HU, в венозную — до 73–93 HU, в отсроченную плотность составила 57 HU. В левом надпочечнике визуализировано новообразование размером 20х14,3х14 мм, с плотностью до 30–49 HU, с накоплением контрастного вещества в артериальную фазу до 77–95 HU, в венозную до 85–94 HU, в отсроченную 50 HU.

Повторно дистанционно консультирован консилиумом врачей-эндокринологов, хирургов и рентгенологов КВМТ СПбГУ. В связи с отсутствием убедительных данных за ФХЦ обоих надпочечников, с целью решения вопроса об оперативном лечении, рекомендовано повторное определение экскреции метаболитов катехоламинов, а также выполнение позитронно-эмиссионной томографии, совмещенной с КТ с 18F-фтордезоксиглюкозой (ПЭТ/КТ с 18F-ФДГ).

При ПЭТ-КТ с 18F-ФДГ новообразования надпочечников имели ровные четкие контуры, округлую форму, размеры определены как 16х16 мм справа и 16х15 мм слева, с неинтенсивным накоплением радиофармпрепарата с SUV 3,35 справа и SUV 4,24 слева, что расценено как признаки, характерные для аденом. Тем не менее при дополнительном контроле в суточной моче экскреция общих нефракционированных метанефринов была повышена до 1463,42 нмоль/с (до 1006,36).

Несмотря на сомнительные результаты визуализирующих исследований, высокая нативная плотность новообразований надпочечников и трижды повышенные уровни метанефринов не позволяли исключить двустороннюю ФХЦ, в связи с чем было рекомендовано оперативное лечение в объеме односторонней адреналэктомии. Амбулаторно проводилась предоперационная подготовка доксазозином с достижением дозировки 4 мг 2 раза в день, на фоне чего уровень АД не превышал 135 и 95 мм рт.ст., пульс — менее 80 ударов в минуту.

При поступлении в Клинику высоких медицинских технологий им. Н.И. Пирогова Санкт-Петербургского государственного университета (КВМТ СПбГУ) при объективном осмотре рост был 170 см, вес — 60 кг, уровень АД — 130 и 70 мм рт.ст., пульс — 70 ударов в минуту. На теле пациента определялись неяркие единичные округлые пятна различного диаметра цвета «кофе с молоком», пятна по типу «веснушчатых гроздьев» в подмышечных впадинах. При активном расспросе пациент сообщил о наличии схожих пятен на коже у матери и бабушки. Клинические признаки позволили заподозрить семейный НФ1, новообразования надпочечников при котором, наиболее вероятно, являются ФХЦ.

Пациенту выполнена однопортовая ретроперитонеоскопическая резекция левого надпочечника с оставлением примерно половины ткани неизмененного надпочечника. По результатам гистологического исследования подтверждена ФХЦ левого надпочечника без признаков сосудистой и капсулярной инвазии. Пациент выписан на 3-и сутки после оперативного лечения в удовлетворительном состоянии, с нормотензией без гипотензивной терапии, без клинических и лабораторных признаков надпочечниковой недостаточности, под динамическое наблюдение хирурга, эндокринолога и онколога. Решение вопроса об оперативном лечении правого надпочечника планируется через 3 месяца по результатам динамического наблюдения.

## Клинический случай №4

Пациентка П., 33 года, поступила в Клинику высоких медицинских технологий им. Н.И. Пирогова Санкт-Петербургского государственного университета (КВМТ СПбГУ) с жалобами на приступы учащенного сердцебиения, сопровождающиеся общей слабостью, продолжительностью от 1 до 5 минут, купирующиеся самостоятельно, а также частые перемены настроения.

Из анамнеза известно, что наблюдается по поводу НФ1. Диагноз был установлен клинически у матери, в связи с чем при появлении характерных кожных проявлений у пациентки носительство мутаций в гене NF1 не вызывало сомнений. Мать умерла от несчастного случая. Также из семейного анамнеза известно, что у брата подобных фенотипических проявлений нет. У пациентки есть дочь 4 лет, которой было проведено генетическое исследование, по результатам которого мутаций в гене NF1 выявлено не было.

При МРТ головного мозга и всего позвоночника данных за патологию получено не было. При МРТ ОБП выявлено новообразование левого надпочечника размером до 41х37х40 мм, с четкими ровными контурами, с признаками контрастного усиления, прилежащее к селезенке, к селезеночной и почечной венам, к левой почке, без признаков инвазии.

По лабораторным результатам обследования, проведенного для оценки гормональной активности надпочечников, отмечалось увеличение экскреции свободных метанефринов до 78,4 мкг в сутки (2,9–52,9) и свободных норметанефринов до 833 мкг в сутки (5,7–67,7). Также было выявлено повышение уровней норадреналина крови до 1370 пг/мл (70–750) и дофамина до 124 пг/мл (до 87), при нормальном уровне адреналина — 50 пг/мл (до 110) и кортизола — 589 нмоль/л (до 650). Уровень электролитов крови находился в пределах референсных значений. По результатам пробы с 1 мг дексаметазона получено полное подавление уровней кортизола крови до 38 нмоль/л и АКТГ менее 5 пг/мл.

Пациентка была консультирована хирургом Клиники высоких медицинских технологий им. Н.И. Пирогова Санкт-Петербургского государственного университета (КВМТ СПбГУ), рекомендовано плановое оперативное лечение. С целью предоперационной подготовки в течение полутора месяцев получала терапию доксазозином 2 мг и метопрололом 1,25 мг в сутки. На этом фоне отмечалось урежение частоты приступов тахикардии, уровень АД составил 90–110/60–70 мм рт.ст., пульс в покое 65–80 ударов в минуту. В связи с наличием в анамнезе хронического гастрита, риском развития катехоламинового криза при проведении фиброгастродуоденоскопии, дополнительно до оперативного лечения пациентка получала рабепразол 20 мг в сутки.

При поступлении в Клинику высоких медицинских технологий им. Н.И. Пирогова Санкт-Петербургского государственного университета (КВМТ СПбГУ) объективно уровень АД составил 110/70 мм рт.ст., пульс 72 удара в минуту. При осмотре по всему телу пациентки отмечались множественные кожные нейрофибромы диаметром до 7 мм, несколько пятен цвета «кофе с молоком» на бедрах от 0,5 см до 2,5 см, «веснушчатые грозди» в подмышечных впадинах.

Выполнена КТ ОБП, при которой отмечено увеличение в динамике размеров многоузлового новообразования левого надпочечника до 44х38х56 мм, нативной плотностью 43 HU, с накоплением контрастного вещества в артериальную фазу до 88 HU, в венозную до 101 HU. Через 10 минут после введения контрастного вещества плотность образования снижалась до 70 HU.

Проведена однопортовая ретроперитонеоскопическая адреналэктомия слева. По результатам гистологического исследования подтверждена ФХЦ левого надпочечника. Пациентка выписана на третьи сутки, послеоперационный период без особенностей.

При контроле в течение года данных за рецидив ФХЦ получено не было. Учитывая наследственную предрасположенность к двустороннему поражению надпочечников, рекомендовано пожизненное динамическое наблюдение.

Сводные данные по описанным клиническим случаям представлены в таблице 1.

**Table:** Таблица 1. Сводные данные по описанным в статье клиническим случаям АЭ — адреналэктомия, резекция — резекция надпочечника, SARA — однопортовый ретроперитонеоскопический доступ, CORA — трехпортовый ретроперитонеоскопический доступ.

№ п/п	Пол	Возраст	Семейный анамнез	Артериальная гипертензия	Сторона поражения надпочечника	Максимальный размер опухоли	Метанефрины суточной мочи	Преимущественный тип катехоламиновой секреции	Хромогранин А в крови	Кортизол после 1 мг дексаметазона	Хирургический доступ	Объем операции	Надпочечниковая недостаточность после операции
1	ж	58	Нет	Да	Левый	61 мм	Повышены	Адреналиновый	Нет данных	Не подавлен	SARA	АЭ	Постоянная
2	ж	22	Да	Нет	Правый	31 мм	Повышены	Адреналиновый	Повышен	Нет данных	CORA	АЭ	Нет
3	м	34	Да	Да	Оба	20 мм	Повышены	Адреналиновый	Норма	Подавлен	SARA	резекция	Нет
4	ж	33	Да	Нет	Левый	56 мм	Повышены	Норадреналиновый	Нет данных	Подавлен	SARA	АЭ	Нет

## Обсуждение

В представленной работе приведено описание четырех клинических случаев ФХЦ в составе семейного НФ1. Считается, что практически у 50% больных мутация в гене NF1 является спорадической [7]. В продемонстрированных нами двух случаях (№2 и №4) было известно об отягощенном семейном анамнезе и о наличии клинических признаков наследственного синдрома, в связи с чем ФХЦ была выявлена при целенаправленных визуализирующих исследованиях.

При активном расспросе у пациента №3 также было установлено наличие кожных проявлений НФ1 по материнской линии, однако обследования с целью выявления патогномоничной для наследственного синдрома родственники не проходили. Вероятной причиной трудностей в диагностике генетической мутации NF1 является стертость клинической картины, заключающейся в малом количестве пятен цвета «кофе с молоком» и неярко выраженной «веснушчатости» только в подмышечных впадинах, с отсутствием кожных нейрофибром и гамартром радужки. Тем не менее, несмотря на столь невыраженные кожные проявления, у больного была выявлена двусторонняя ФХЦ. В литературе встречаются данные о выявлении мутации гена NF1 при наличии катехоламин-продуцирующей опухоли надпочечника даже у пациентов без характерных клинических проявлений наследственного синдрома, что демонстрирует необходимость проведения генетического тестирования [24].

Единственным из представленных случаев с невыявленной наследственностью была пациентка №1, однако следует отметить, что она могла сообщить только об анамнезе со стороны матери, в связи с чем нельзя исключить возможное наследование по отцовской линии.

Примечательно, что у обеих пациенток (№2 и №4) с известной отягощенной наследственностью по НФ1 отсутствовало повышение уровня АД. Считается, что для клинической картины ФХЦ/ПГ характерно кризовое повышение уровня систолического АД до 200–250 мм рт.cт., сопровождающееся чувством страха, дрожью в теле, учащенным сердцебиением, профузным потоотделением. Однако на практике данное сочетание симптомов встречается не более чем у 25% пациентов [7]. Подобная классическая симптоматика отмечалась только в первом клиническом случае, ассоциированном с увеличением экскреции метанефринов более чем в 10 раз, что подтверждает редкость встречаемости классических жалоб ФХЦ, особенно при наследственных синдромах.

По оценкам разных авторов, распространенность бессимптомных форм ФХЦ при НФ1 составляет от 22 до 80%, что связано, с одной стороны, с малочисленностью описываемых выборок, а с другой — с по-прежнему высоким числом ФХЦ, выявляемых на аутопсиях [2][17][18].

В клиническом случае №2 пациентка Ш. 22 лет не предъявляла никаких жалоб, тогда как в клиническом случае №4 у пациентки П. 33 лет присутствовали пароксизмальные симптомы в виде кратковременных эпизодов учащенного сердцебиения. Интересно, что отсутствие такого патогномоничного симптома ФХЦ, как АГ, не коррелировало с размером новообразования надпочечника или уровнем катехоламинов. Так, в малосимптомном случае №4 размер ФХЦ был даже больше, чем при развернутой клинической симптоматике у пациентки №1, составив 56 и 49 мм соответственно. В случае №2 отсутствовали жалобы, несмотря на повышение экскреции метанефринов с суточной мочой в 2,5 раза, а у пациентки №4 невыраженная клиническая симптоматика ассоциировалась с увеличением экскреции норметанефринов с суточной мочой более чем в 10 раз.

В случае №1 ФХЦ была выявлена при обследовании с целью исключения вторичных причин АГ. У пациента №3 новообразования надпочечников были выявлены случайно, при обследовании по поводу боли в поясничной области. В работе Shinall M.C. и соавт. представлены данные 56 больных с ФХЦ, среди которых у 6 пациентов был выявлен НФ1. У всех шести пациентов опухоль надпочечника была выявлена случайно, при этом лишь у 1 больного отмечалась мягкая АГ, а также жалобы на головную боль и ощущение учащенного сердцебиения [25]. Эти данные в совокупности с представленными нами случаями демонстрируют необходимость скрининга на ФХЦ у пациентов с НФ1 не только при повышении уровня АД, как рекомендует ERN GENTURIS, но и в отсутствие патогномоничных симптомов.

Большинство ФХЦ являются спорадическими односторонними. Частота встречаемости двусторонних новообразований составляет 7–10% [26]. При семейных синдромах встречаемость синхронных или метахронных двусторонних поражений мозгового слоя надпочечников значительно возрастает [7]. В представленной выборке в трех случаях было выявлено одностороннее поражение надпочечников, тогда как в одном — одновременно двусторонний процесс.

Различались типы гормональной секреции представленных ФХЦ в составе НФ1. В представленных случаях №1, №2 и №3 отмечались преимущественно адреналин-секретирующие опухоли надпочечников, тогда как №4 — преимущественно норадреналиновый тип гормональной секреции новообразования (табл. 1). Эти наблюдения соответствуют данным литературы, показавшим возможность секреции ФХЦ при НФ1 как адреналина, так и норадреналина [7].

Различалась информативность анализа крови на хромогранин А в диагностике ФХЦ при НФ1. В случае №2 уровень хромогранина А был повышен до 120,2 мкг/л (до 100), при этом у пациента №3 его концентрация находилась в пределах референсных значений — 46,37 мкг/л (до 100). У больных №1 и №4 данный анализ не исследовался.

По результатам исследования глюкокортикоидной секции убедительных данных за ко-секрецию получено не было, однако в первом описанном нами случае уровень кортизола после ночного подавляющего теста с 1 мг дексаметазона составил 106,8 нмоль/л, что соответствовало «серой зоне» показателя, то есть свидетельствовало о возможной автономной ко-секреции кортизола. У пациентов №3 и №4 уровень кортизола после данной пробы был полностью подавлен, таким образом, органический гиперкортицизм был исключен. В третьем случае тест не проводился, однако фоновые уровни кортизола в крови и суточной моче были в пределах референсных значений. Примечательно, что послеоперационная надпочечниковая недостаточность развилась именно у пациентки с ФХЦ с возможной автономной ко-секрецией кортизола, что косвенно может свидетельствовать в пользу подтверждения имевшейся сочетанной гормональной продукции опухолью с развитием истончения коркового слоя второго надпочечника.

В исследовании Shinall M.C. и соавт. размеры ФХЦ в группе пациентов с НФ1 были достоверно меньше по сравнению со спорадической формой заболевания, составив 2,75 см против 5,9 см (p=0,014). Кроме того, значимо меньше была частота послеоперационных осложнений [24]. В представленной нами работе размеры ФХЦ варьировали от 18 до 40 мм на момент первичной визуализации. При этом обращает на себя внимание быстрый рост новообразований, который возможно отследить в трех случаях отложенного оперативного лечения. Так, у пациентки №1 58 лет с момента первичной диагностики до оперативного лечения через 7 месяцев (в связи с личными обстоятельствами) ФХЦ, по данным КТ, увеличилась с 49 до 61 мм, а у пациентки №4 33 лет за 2 месяца гормонального обследования инциденталомы надпочечника и предоперационной подготовки доксазозином — с 41 до 56 мм. О быстром росте ФХЦ можно судить и в случае №2, при котором максимальный размер новообразования на момент первичной диагностики по КТ составлял 31 мм, а по результатам гистологического исследования макропрепарата после оперативного лечения, выполненного по семейным обстоятельствам только через 6 месяцев, — до 50 мм.

Всем представленным в статье пациентам проведено оперативное лечение надпочечников. Согласно национальным клиническим рекомендациям, методикой выбора является эндоскопическое удаление ФХЦ, а при новообразованиях более 8 см и/или инвазивных опухолях рекомендован открытый доступ во избежание повреждения капсулы опухоли и ее диссеминирования [12]. В КВМТ СПбГУ применяется ретроперитонеоскопическая методика адреналэктомии, обладающая рядом преимуществ по сравнению с лапароскопическим доступом в виде уменьшения интраоперационной кровопотери, потребности в анальгетиках и продолжительности госпитализации, а также отсутствием необходимости рассечения спаек при наличии спаечного процесса в брюшной полости [27]. В случаях №1, №3 и №4 применялась однопортовая модификация ретроперитонеоскопического доступа, которая, как и трехпортовый вариант, может быть выполнена при размере опухоли до 8 см при наличии технической возможности, дополнительно позволяющая снизить болевой синдром и достичь лучшего косметического эффекта по сравнению с традиционным трехпортовым доступом, однако требующая более высокой квалификации и опыта хирурга [28–30].

Дискутабельным является вопрос о резекции надпочечников при наследственной ФХЦ. С одной стороны, удаление одного надпочечника у большинства пациентов не приводит к снижению выработки глюкокортикоидных и минералкортикоидных гормонов, что продемонстрировано в случаях №3 и №4. С другой стороны, развитие надпочечниковой недостаточности все же возможно, особенно при наличии ко-секреции кортизола, что показано в случае №1. Двустороннее оперативное лечение надпочечников неизбежно приводит к необходимости пожизненного приема заместительной гормональной терапии. Первичная надпочечниковая недостаточность ассоциирована с риском возникновения гипоадреналового криза, преимущественно в состоянии острого стресса, а также с развитием ятрогенного синдрома Иценко-Кушинга у стероидзависимых пациентов [31].

Органосохраняющие операции у пациентов с наследственным синдромом ассоциированы с увеличением частоты рецидивов, о чем должны быть предупреждены пациенты, однако в исследованиях показано, что при этом не возрастает частота метастатического поражения и не снижается выживаемость больных [32]. Кроме того, показано, что риск ипсилатерального рецидива значительно ниже по сравнению с контралатеральным (0–14% против 43–57% соответственно) [33], в связи с чем резекция надпочечников рассматривается как одна из опций оперативного лечения с целью предотвращения развития как хронической, так и острой надпочечниковой недостаточности, улучшения качества жизни пациентов. Исключением является носительство наследственных мутаций, ассоциированных с высоким риском метастазирования ФХЦ, к которым относятся SDHB, SDHD, MAX, TMEM127, HRAS, CSDE1 и MAML3, в связи с чем выполнение органосохраняющих операций в данных случаях не рекомендовано [27].

Интересным является факт обнаружения мутации в гене RET, гетерозиготного варианта p.His926Leu с неизвестным клиническим значением, в случае №1. Подобные неожиданные сочетания мутаций различных генов, в том числе ассоциированных с ФХЦ, описаны в литературе. Так, в работе Mellid S. и соавт. из 23 пациентов с ФХЦ/ПГ в рамках синдрома НФ1 у 3 были выявлены герминальные мутации в генах DLST (n=1) и MDH2 (n=2), у 2 — соматические мутации в генах H3F3A и PRKAR1A [34].

## Заключение

НФ1 является наследственным заболеванием с аутосомно-доминантным типом наследования, в связи с чем необходимо генетическое обследование всех родственников первой линии родства с целью раннего выявления синдрома и ассоциированных с ним заболеваний. ФХЦ/ПГ при носительстве мутации в гене NF1 может встречаться, даже при отсутствии подобных фенотипических проявлений у родителей с синдромом НФ1. Учитывая высокую частоту бессимптомного течения ФХЦ при НФ1, целесообразно регулярное профилактическое обследование с целью раннего выявления новообразований надпочечников. Эндоскопические операции являются лечением выбора при выявлении ФХЦ/ПГ, при этом рядом преимуществ обладает ретроперитонеоскопическая методика. При размере новообразований надпочечников менее 8 см и наличии необходимого опыта у хирурга возможно применение миниинвазивного однопортового доступа. Резекция надпочечников является дискутабельным вариантом лечения ФХЦ при НФ1, требует индивидуального подхода, обсуждения с пациентом, с взвешенной оценкой преимуществ отсутствия или снижения дозировок/кратности приема заместительной гормональной терапии и недостатков в виде риска рецидивов и, хоть минимального, но все же описанного в литературе риска метастазирования.

## Дополнительная информация

Источники финансирования. Работа выполнена по инициативе авторов без привлечения финансирования.

Конфликт интересов. Авторы декларируют отсутствие явных и потенциальных конфликтов интересов, связанных с содержанием настоящей статьи.

Участие авторов.Все авторы одобрили финальную версию статьи перед публикацией, выразили согласие нести ответственность за все аспекты работы, подразумевающую надлежащее изучение и решение вопросов, связанных с точностью или добросовестностью любой части работы.

Согласие пациента. Пациенты добровольно подписали информированное согласие на публикацию персональной медицинской информации в обезличенной форме.

## References

[cit1] Williams Virginia C., Lucas John, Babcock Michael A., Gutmann David H., Korf Bruce, Maria Bernard L. (2008). Neurofibromatosis Type 1 Revisited. Pediatrics.

[cit2] Stewart Douglas R., Korf Bruce R., Nathanson Katherine L., Stevenson David A., Yohay Kaleb (2018). Care of adults with neurofibromatosis type 1: a clinical practice resource of the American College of Medical Genetics and Genomics (ACMG). Genetics in Medicine.

[cit3] Miraglia E, Moliterni E, Iacovino C, et al. Cutaneous manifestations in neurofibromatosis type 1. Clin Ter. 2020;171(5):e371-e377. doi: https://doi.org/10.7417/CT.2020.2242 32901776

[cit4] Shofty Ben, Barzilai Ori, Khashan Morsi, Lidar Zvi, Constantini Shlomi (2020). Spinal manifestations of Neurofibromatosis type 1. Child's Nervous System.

[cit5] Yukina M. Yu., Avsievich E. S., Pushkareva A. S., Nuralieva N. F., Bondarenko E. V., Platonova N. M., Beltsevich D. G., Troshina E. A. (2022). Atypical and typical course of neurofibromatosis type 1 in combination with pheochromocytoma. Endocrine Surgery.

[cit6] Gutmann David H., Ferner Rosalie E., Listernick Robert H., Korf Bruce R., Wolters Pamela L., Johnson Kimberly J. (2017). Neurofibromatosis type 1. Nature Reviews Disease Primers.

[cit7] Rebrova D. V., Vorokhobina N. V., Imyanitov E. N., Rusakov V F., Krasnov L. M., Sleptsov I. V., Chernikov R. A., Fedorov E. A., Semenov A. A., Chinchuk I. K., Sablin I. V., Alekseev M. A., Kuleshov O. V., Fedotov Ju. N (2022). Clinical and laboratory features of hereditary pheochromocytoma and paraganglioma. Problems of Endocrinology.

[cit8] Saleh Mustafa, Dib AlFadel, Beaini Sarah, Saad Charbel, Faraj Sary, El Joueid Youssef, Kotob Yasmine, Saoudi Lara, Emmanuel Nancy (2023). Neurofibromatosis type 1 system-based manifestations and treatments: a review. Neurological Sciences.

[cit9] (2015). Neurofibromatosis type 1. Handbook of Clinical Neurology.

[cit10] Aronow Mary E., Wiley Henry E., Gaudric Alain, Krivosic Valerie, Gorin Michael B., Shields Carol L., Shields Jerry A., Jonasch Eric W., Singh Arun D., Chew Emily Y. (2019). VON HIPPEL–LINDAU DISEASE. Retina.

[cit11] Kehrer-Sawatzki Hildegard, Cooper David N. (2021). Challenges in the diagnosis of neurofibromatosis type 1 (NF1) in young children facilitated by means of revised diagnostic criteria including genetic testing for pathogenic NF1 gene variants. Human Genetics.

[cit12] Mel'nichenko G.A. i soavt. Klinicheskie rekomendatsii Rossiiskoi assotsiatsii endokrinologov po diagnostike i lecheniyu feokhromotsitomy/paragangliomy. // Endokrinnaya khirurgiya. — 2015. — T.9. — №3. — S.15-33. doi: https://doi.org/10.14341/serg201531533

[cit13] Garcia-Carbonero R., Matute Teresa F., Mercader-Cidoncha E., Mitjavila-Casanovas M., Robledo M., Tena I., Alvarez-Escola C., Arístegui M., Bella-Cueto M. R., Ferrer-Albiach C., Hanzu F. A. (2021). Multidisciplinary practice guidelines for the diagnosis, genetic counseling and treatment of pheochromocytomas and paragangliomas. Clinical and Translational Oncology.

[cit14] Loponen Niina, Ylä‐Outinen Heli, Kallionpää Roope A., Valtanen Mikko, Auranen Kari, Järveläinen Hannu, Peltonen Sirkku, Peltonen Juha (2023). Hypertension in NF1: A closer look at the primacy of essential hypertension versus secondary causes. Molecular Genetics & Genomic Medicine.

[cit15] Zinnamosca Laura, Petramala Luigi, Cotesta Dario, Marinelli Cristiano, Schina Mauro, Cianci Rosario, Giustini Sandra, Sciomer Susanna, Anastasi Emanuela, Calvieri Stefano, De Toma Giorgio, Letizia Claudio (2010). Neurofibromatosis type 1 (NF1) and pheochromocytoma: prevalence, clinical and cardiovascular aspects. Archives of Dermatological Research.

[cit16] Tachibana Akira, Iida Kota, Itami Yoshitaka, Hashimura Masaya, Hosokawa Yukinari, Fujimoto Kiyohide (2023). Composite pheochromocytoma associated with neurofibromatosis type 1. IJU Case Reports.

[cit17] Dupuis Hippolyte, Chevalier Benjamin, Cardot-Bauters Catherine, Jannin Arnaud, Do Cao Christine, Ladsous Miriam, Cortet Christine, Merlen Emilie, Drouard Magali, Aubert Sébastien, Vidaud Dominique, Espiard Stéphanie, Vantyghem Marie-Christine (2023). Prevalence of Endocrine Manifestations and GIST in 108 Systematically Screened Patients With Neurofibromatosis Type 1. Journal of the Endocrine Society.

[cit18] Képénékian Lori, Mognetti Thomas, Lifante Jean-Christophe, Giraudet Anne-Laure, Houzard Claire, Pinson Stéphane, Borson-Chazot Françoise, Combemale Patrick (2016). Interest of systematic screening of pheochromocytoma in patients with neurofibromatosis type 1. European Journal of Endocrinology.

[cit19] Kiriakopoulos Andreas, Giannakis Periklis, Menenakos Evangelos (2023). Pheochromocytoma: a changing perspective and current concepts. Therapeutic Advances in Endocrinology and Metabolism.

[cit20] Farias Francisco, Yogeswaran Vidhushei, Hidano Danelle, Starnes Elizabeth, Kwon Young, Branch Kelley, Tylee Tracy, Poole Jeanne, Sridhar Arun (2023). Ventricular fibrillation due to pheochromocytoma crisis in a previously asymptomatic patient. Journal of Cardiology Cases.

[cit21] Petr Elisabeth Joye, Else Tobias (2018). Pheochromocytoma and Paraganglioma in Neurofibromatosis type 1: frequent surgeries and cardiovascular crises indicate the need for screening. Clinical Diabetes and Endocrinology.

[cit22] Bausch B., Borozdin W., Neumann H.P. Clinical and genetic characteristics of patients with neurofibromatosis type 1 and pheochromocytoma. N Engl J Med. 2006;354(25):2729–2731 16790714 10.1056/NEJMc066006

[cit23] Carton Charlotte, Evans D. Gareth, Blanco Ignacio, Friedrich Reinhard E., Ferner Rosalie E., Farschtschi Said, Salvador Hector, Azizi Amedeo A., Mautner Victor, Röhl Claas, Peltonen Sirkku, Stivaros Stavros, Legius Eric, Oostenbrink Rianne, Brunet Joan, Van Calenbergh Frank, Cassiman Catherine, Czech Thomas, Gavarrete de León María José, Giele Henk, Henley Susie, Lazaro Conxi, Lipkovskaya Vera, Maher Eamonn R., Martin Vanessa, Mathijssen Irene, Opocher Enrico, Pires Ana Elisabete, Pletschko Thomas, Poupaki Eirene, Ridola Vita, Rietman Andre, Rosenbaum Thorsten, Santhouse Alastair, Sehested Astrid, Simmons Ian, Taal Walter, Wagner Anja (2023). ERN GENTURIS tumour surveillance guidelines for individuals with neurofibromatosis type 1. eClinicalMedicine.

[cit24] Buffet Alexandre, Burnichon Nelly, Favier Judith, Gimenez-Roqueplo Anne-Paule (2020). An overview of 20 years of genetic studies in pheochromocytoma and paraganglioma. Best Practice & Research Clinical Endocrinology & Metabolism.

[cit25] Shinall Myrick C, Solórzano Carmen C (2014). Pheochromocytoma in Neurofibromatosis Type 1: When Should it Be Suspected. Endocrine Practice.

[cit26] Lider Burciulescu Sofia Maria, Gheorghiu Monica Livia, Muresan Andrei, Gherlan Iuliana, Patocs Attila, Badiu Corin (2024). Bilateral pheochromocytomas: clinical presentation and morbidity rate related to surgery technique and genetic status. Endocrine Connections.

[cit27] Shikhmagomedov Sh. Sh., Rebrova D. V., Krasnov L. M., Fedorov E. A., Chinchuk I. K., Chernikov R. A., Rusakov V. F., Slepstov I. V., Zgoda E. A. (2023). Surgical treatment of pheochromocytoma. Problems of Endocrinology.

[cit28] Walz Martin K., Alesina Piero F., Wenger Frank A., Deligiannis Anastasios, Szuczik Eduard, Petersenn Stephan, Ommer Andreas, Groeben Harald, Peitgen Klaus, Janssen Onno E., Philipp Thomas, Neumann Hartmut P.H., Schmid Kurt W., Mann Klaus (2006). Posterior retroperitoneoscopic adrenalectomy—results of 560 procedures in 520 patients. Surgery.

[cit29] Shikhmagomedov Sh. Sh., Rebrova D. V., Alekseev M. A., Krasnov L. M., Fedorov E. A., Chinchuk I. K., Chernikov R. A., Rusakov V. F., Sleptsov I. V., Sablin I. V., Kuleshov O. V. (2024). Comparison of intraoperative hemodynamics in classical and single-port adrenalectomy. Endocrine Surgery.

[cit30] Luo Yancheng, Chen Xiang, Chen Zhi, He Yao, Li Nannan, Lai Cheng, Xie Chaoqun (2012). Retroperitoneal Laparoendoscopic Single-Site Adrenalectomy: Our Initial Technical Experience. Journal of Laparoendoscopic & Advanced Surgical Techniques.

[cit31] Neumann Hartmut P. H., Tsoy Uliana, Bancos Irina, Amodru Vincent, Walz Martin K., Tirosh Amit, Kaur Ravinder Jeet, McKenzie Travis, Qi Xiaoping, Bandgar Tushar, Petrov Roman, Yukina Marina Y., Roslyakova Anna, van der Horst-Schrivers Anouk N. A., Berends Annika M. A., Hoff Ana O., Castroneves Luciana Audi, Ferrara Alfonso Massimiliano, Rizzati Silvia, Mian Caterina, Dvorakova Sarka, Hasse-Lazar Kornelia, Kvachenyuk Andrey, Peczkowska Mariola, Loli Paola, Erenler Feyza, Krauss Tobias, Almeida Madson Q., Liu Longfei, Zhu Feizhou, Recasens Mònica, Wohllk Nelson, Corssmit Eleonora P. M., Shafigullina Zulfiya, Calissendorff Jan, Grozinsky-Glasberg Simona, Kunavisarut Tada, Schalin-Jäntti Camilla, Castinetti Frederic, Vlcek Petr, Beltsevich Dmitry, Egorov Viacheslav I., Schiavi Francesca, Links Thera P., Lechan Ronald M., Bausch Birke, Young William F., Eng Charis (2019). Comparison of Pheochromocytoma-Specific Morbidity and Mortality Among Adults With Bilateral Pheochromocytomas Undergoing Total Adrenalectomy vs Cortical-Sparing Adrenalectomy. JAMA Network Open.

[cit32] Zawadzka Karolina, Tylec Piotr, Małłczak Piotr, Major Piotr, Pe¸dziwiatr Michał, Pisarska-Adamczyk Magdalena (2022). Cortical-sparing adrenalectomy for bilateral pheochromocytoma - is it a game worth the candle? Systematic review with meta-analysis comparing total vs partial adrenalectomy in bilateral pheochromocytoma. Endocrine Abstracts.

[cit33] Perysinakis Iraklis, Aggeli Ch., Kaltsas Gr., Zografos G. N. (2020). Adrenal-sparing surgery: current concepts on a theme from the past. Hormones.

[cit34] Mellid Sara, Gil Eduardo, Letón Rocío, Caleiras Eduardo, Honrado Emiliano, Richter Susan, Palacios Nuria, Lahera Marcos, Galofré Juan C., López-Fernández Adriá, Calatayud Maria, Herrera-Martínez Aura D., Galvez María A., Matias-Guiu Xavier, Balbín Milagros, Korpershoek Esther, Lim Eugénie S., Maletta Francesca, Lider Sofia, Fliedner Stephanie M. J., Bechmann Nicole, Eisenhofer Graeme, Canu Letizia, Rapizzi Elena, Bancos Irina, Robledo Mercedes, Cascón Alberto (2023). Co-occurrence of mutations in NF1 and other susceptibility genes in pheochromocytoma and paraganglioma. Frontiers in Endocrinology.

